# Towards a medical school curriculum for uncertainty in clinical practice

**DOI:** 10.1080/10872981.2021.1972762

**Published:** 2021-08-29

**Authors:** Dimitrios Papanagnou, Nethra Ankam, David Ebbott, Deborah Ziring

**Affiliations:** a Professor and Vice Chair for Education in the Department of Emergency Medicine and Associate Dean for Faculty Development, Sidney Kimmel Medical College at Thomas Jefferson University, Philadelphia, PA, USA; b Associate Professor in the Department of Rehabilitation Medicine, Sidney Kimmel Medical College at Thomas Jefferson University, Philadelphia, PA, USA; c Third-year medical student, Sidney Kimmel Medical College at Thomas Jefferson University, Philadelphia, PA, USA; d Clinical Associate Professor in the Department of Medicine and Senior Associate Dean for Academic Affairs, Sidney Kimmel Medical College at Thomas Jefferson University, Philadelphia, PA, USA

**Keywords:** Undergraduate medical education, uncertainty, curriculum design, well-being, self-efficacy, humanities

## Abstract

Uncertainty abounds in the clinical environment. Medical students, however, are not explicitly prepared for situations of uncertainty in clinical practice, which can cause anxiety and impact well-being. To address this gap, we sought to capture how students felt in various clinical scenarios and identify programs they found helpful as they worked through uncertainty in their clerkships to better inform curriculum that prepares them to acknowledge and navigate this uncertainty. This is an observational cross-sectional study of third-year medical students surveyed at the end of core clerkships. The survey consisted of the General Self-Efficacy (GSE) Scale and Intolerance of Uncertainty Scale (IUS). Items asked students to rate preparedness, confidence, and comfort with uncertainty in clinical practice. Items on curricular programs asked students to identify training that prepared them for uncertainty in clerkships, and examined correlations with specific clinical practice uncertainty domains (CPUDs). Spearman’s rank-order correlation, Chi-Square, and ANOVA were used to analyze quantitative data. Open responses were analyzed using Braun and Clarke’s Framework. Response rate was 98.9% (287/290). GSE was inversely correlated with IUS (p < 0.001). GSE was positively correlated with all CPUDs (p < 0.005). IUS had an inverse correlation with all CPUDs (p < 0.005). Pedagogies with statistically-significant relationships with preparing students for uncertainty, communicating and building relationships with patients during times of uncertainty, and overall well-being included: team debriefs, role plays, case- and team-based learning, story slams, and sharing narratives with peers and faculty (p < 0.05). Qualitatively, students appreciated storytelling, role-modeling of communication strategies, debriefing, and simulations. Strategically immersing specific educational formats into formal curriculum may help cultivate skills needed to prepare students for uncertainty. Clinical debriefs, interprofessional role plays, simulations, communications skills training, instructor emotional vulnerability, storytelling, and peer-to-peer conversations may have the most impact. Further study is required to evaluate their longitudinal impact.

## Introduction

For many students, coping with the inherent uncertainties of clinical practice can cause them to struggle as they transition from the classroom to clinical learning environment (CLE). These may include struggles with diagnosis, management, and communication. To date, undergraduate medical education has effectively trained medical students for certainty; but formal training for uncertainty in clinical practice has been lacking in formal curriculum. Well-designed educational programs that specifically address uncertainty have the potential to empower students to thrive during the transition to clinical practice.

Lee et al offer an operational definition for uncertainty, and describe it as ‘the dynamic, subjective perception of not knowing what to think, feel, or do.’[1] Under this definition, three core dimensions of uncertainty in clinical practice have been described – the source of uncertainty, the subjective nature of uncertainty, and responses to uncertainty[1]. Comfort level with uncertainty, which directly links to all of these dimensions, impacts patient care. Specifically, comfort with uncertainty can impact patient communication[2], decision-making ability[3], resource utilization[2], and patient dispositions[4]. Anxiety towards clinical uncertainty is associated with increased cost of care, as well as a reluctance to fully disclose information to patients[2]. Studies have suggested that tolerance to uncertainty impacts willingness to work with underserved communities, and may even influence how trainees address pain management during times of ambiguity [4–6]. These observations further strengthen the case for intentionally focusing on uncertainty in an undergraduate medical education (UME) curriculum.

Unfortunately, formal programs in UME do not address the uncertainty that is inherent in clinical practice. Traditional medical education programs train students for certainty; and, as a result, students become more comfortable with linear thinking early in their training. Students are rewarded for correct answers on examinations and correct diagnoses and treatments during simulations[7]. As an example, question stems and case vignettes prime students to expect that diseases will present uniformly, progress similarly, and respond to treatments accordingly. Thus, a mismatch of student expectations and the realities of clinical uncertainty collide during this transition. Students ill-equipped to address uncertainty in the clinical environment can experience cognitive dissonance[8], diminished self-efficacy, erosion of empathy[9], maladaptive perfectionism[6], and eventual burnout later in their careers[2]. The emphasis on linear thinking can thwart creative problem-solving and the ability to calibrate for uncertainty [9] – skills that are essential to thrive in clinical practice.

Opportunities for curricular development that prepares students for this uncertainty have been described. The case for liberal arts and humanities programs to improve future physician’s abilities to think ‘laterally’ has been asserted by Cristancho[10]. Incorporating cognitive disequilibrium into pre-clinical training through patient-centered narratives has been advocated by Kumagai[11]. Simulations and follow-up discussions of complex patient cases were tested by Scott et al, and proposed that simulations can provide disheartening, yet useful reflections, for students[12]. Recently, Tonelli et al encouraged bringing the ‘philosophy of medicine’ into clinical courses and bedside teaching to develop a clinical uncertainty taxonomy for familiarity and competence[13]. Curricular innovations such as these can help bring uncertainty in clinical practice explicitly to the forefront of medical education training.

From the lens of curriculum development, there is also an opportunity to formally focus on self-efficacy, ‘the confidence to carry-out the courses of action necessary to accomplish desired goals,’ as a means to better prepare students for the uncertainty that exists in the CLE[14]. There has been increasing interest in medical students’ self-efficacy, specifically as it pertains to their learning and development. In most cases, individuals will choose to engage in an activity if they are confident of success, and potentially avoid those activities in which they are not confident. Given the dynamic interplay of environmental and behavioral factors in the clinical environment, self-efficacy may play an important role in influencing learner success[14]. Learning in the clinical environment is dependent on overcoming a range of intellectual, social, and motivational challenges that prompt doubt[14]. While the relationship between self-efficacy and tolerance of ambiguity has been explored[15], the literature does not describe whether students with higher self-efficacy are more tolerant of uncertainty. A study of five medical schools investigating the effects of a humanities curriculum demonstrated a statistically-significant relationship between Generalized Self Efficacy and Budner’s Tolerance of Ambiguity scales[15]. If this relationship can be further clarified, a case can be made to formally develop curriculum that can help address student self-efficacy.

Our study explores the role a pre-clinical medical education curriculum based in case-based learning and the humanities can have on clinical uncertainty in medical students when they enter clerkships. We aimed to capture how students felt in various clinical scenarios, and identify what types of educational programs they found helpful as they worked through the clinical uncertainty in their clerkships. Specifically, we sought to: 1) describe perceived comfort with uncertainty encountered across clerkships; 2) identify curricular elements that best prepared students for these situations; and 3) solicit suggestions from students that would have better prepared them for this uncertainty. We hypothesized that certain training components will correlate with clinical uncertainty comfort, and themes will emerge from free responses to guide longitudinal curricular design and instruction, as well as preparation for practice in the clinical learning environment.

## Methods

### Study design

This was a descriptive, cross-sectional study that employed a survey-based design using target sampling to collect data through an online link. The authors intentionally selected this methodology, as they wanted to capture student experiences at the end of their year-long clinical clerkship experiences. The survey instrument was a 30-question, anonymous, electronic questionnaire (included in the Appendix). No incentives were offered for completion of the questionnaire. Both quantitative and qualitative data were collected. Students had two weeks to complete the survey. An email remainder was sent to students one week after the initial invitation to participate. The study was reviewed and approved by the institutional review board (IRB) of the University for the involvement of human subjects (#20E.805).

### Participants

Two hundred ninety third-year medical students (Class of 2021) at an urban medical school in Philadelphia, Pennsylvania, USA were invited to participate in the survey via email within two weeks after completing all third-year core clerkships in April 2020. The medical school curriculum takes place over four years: the first two years of training are comprised of traditional pre-clinical coursework, after which students begin their third year of training as they transition into the clinical environment (i.e., in the form of core clerkship experiences). Students were surveyed immediately after completing all core clerkship requirements to capture their experiences with uncertainty in clinical practice over the course of their year-long clinical immersion. Completed core clerkships included Emergency Medicine, Family Medicine, Internal Medicine, Neurology, Obstetrics and Gynecology, Pediatrics, Psychiatry, Surgery and Surgical Subspecialties. Only students who successfully completed required clerkships by April 2020 were invited to participate; students who did not complete all required clerkships were excluded from participating in the study.

### Instrument

The questionnaire was designed through consensus by the study investigators, who represent experienced educators with training in qualitative research design and educational research methods. An extensive literature review was conducted on uncertainty in health professions education and training, which included studies that employed survey design to better understand the effects uncertainty has on trainees and providers in the clinical practice environment. As no previously validated surveys were identified in the literature, the study investigators applied best practices to develop the current survey [16,17].

Items underwent iterative review, and were reviewed for clarity of both content and structure by the research team. Cycles of feedback from the co-authors were applied to rounds of survey edits [16,17]. The survey consisted of quantitative questions that required respondents to make a discrete selection from listed choices, including the option of ‘other’ with a text clarification box. It also included qualitative data in response to open-ended questions that had unlimited free text entry. The electronic link to the questionnaire was pre-tested for functionality by the investigators prior to distribution to study participants.

### Survey content

The survey consisted of four sections:

Section 1: General Self Efficacy (GSE), a validated GSE Scale developed by Schwarzer and Jerusalem[18]. The GSE scale is a 10-item psychometric questionnaire that measures one’s optimistic beliefs to cope with life’s difficult demands. Since this is a self-reported measure, the instrument measures a perception of self-efficacy in individuals[18]. The scale has been used in numerous research studies, where it typically yielded internal consistency with Cronbach-alpha values ranging between 0.75 and 0.91[19]. Participants are asked to review a series of statements (e.g., ‘It is easy for me to stick to my aims and accomplish my goals’) and indicate their degree of agreement with each item on a four-point Likert scale (i.e., not at all true, hardly true, moderately true, exactly true). Composite scores for GSE range from 10 (low GSE) to 40 (high GSE). The frequency distribution of self-efficacy sum scores in sampled populations approximates a normal distribution (mean 29.55 and standard deviation 5.32)[19].

Section 2: Intolerance to Uncertainty (IUS), a validated scale to gauge intolerance to uncertainty, Short Form Version, developed by Carleton, Norton, and Asmundson[20]. The IUS scale, short form, is a 12-item instrument that measures reactions to uncertainty, ambiguous situations, and the future (e.g., “I always want to know what the future has in store for me). The short-form scale is based on the original 27-item IUS scale and has the same internal consistency and convergent validity as the original version[21]. Participants are asked to review a series of statements and indicate their level of agreement on a 5-point Likert scale, ranging from 1 (not at all characteristic of me) to 5 (entirely characteristic of me). Scores range from 12 (low IUS) to 60 (high IUS). The IUS short-form scale was chosen because of its psychometric comparability to the longer version and its brevity. Items on the scale address prospective anxiety (e.g., ‘I cannot stand being taken by surprise’) and inhibitory anxiety (e.g., ‘When it’s time to act, uncertainty paralyzes me’), both of which have high internal consistencies (Cronbach-alpha, 0.85)[21]. Previous studies with the IUS short-form scale have demonstrated broadly normal distributions in sampled populations, with a community-reported mean of 29.53 (standard deviation 10.96)[21].

Section 3: Comfort with Uncertainty in Clinical Practice. To better understand medical students’ comfort with uncertainty in clinical practice, the authors focused on specific Clinical Practice Uncertainty Domains (CPUD), based on items developed by the authors, and informed by existing conceptual frameworks for uncertainty in clinical practice [1,22]. Survey items for this section were designed through consensus by the study investigators. An extensive literature review was conducted on uncertainty in clinical practice to delineate domains in which uncertainty in the clinical environment affects learners. After several focus groups with the authors, items in this section were grouped into the four following categories:

1. Preparation for uncertainty in clinical practice;
2. Confidence with communicating with patients during times of clinical uncertainty;
3. Forming meaningful relationships with patients during times of clinical uncertainty; and
4. How wellbeing is affected when exposed to clinically uncertain situations.

This section consisted of a series of statements (e.g., ‘I feel prepared to address uncertain situations during clinical clerkships’) and asked respondents to make a discrete selection from a Likert scale of available choices to measure agreement with each statement (i.e., not at all, somewhat, very, or entirely). These items were piloted with a cohort of fourth-year medical students. Items were then reviewed by an expert in survey design (i.e., non-clinician, education researcher) for readability outside of the target audience.

Section 4: Perceptions of a Curriculum’s Ability to Prepare for Uncertainty in Clinical Practice, also based on items developed by the authors, including open-ended, free response items. Survey items for this section were designed through consensus by the study investigators. The authors reviewed the medical school curriculum for programs and pedagogies that participants were exposed to in their first 3 years of training to clarify what specific experiences better prepare students to address uncertainty in the clinical environment. This section consisted of quantitative questions that asked respondents to make a discrete selection from listed choices (i.e., none at all, a little, a moderate amount, a lot, a great deal). Items were piloted with a cohort of fourth-year medical students and were then reviewed by an expert in survey design (i.e., non-clinician, education researcher) for readability outside of the target audience.

### Survey administration

The authors used Qualtrics software (Qualtrics, Provo, UT) to administer the online questionnaire. The electronic link to the questionnaire was tested for functionality by the investigators prior to distribution to study participants. All students in the Class of 2021 received a solicitation email with the request to participate; the electronic link for the survey was included in the email. Email solicitation to complete the survey took place prior to viewing a virtual, pre-recorded lecture on uncertainty in clinical practice 2 weeks before beginning the transition-to-residency course. Investigators emphasized the confidentiality and voluntary nature of the study. Duplicate survey completion by any participant was prevented by disabling this feature on the Web-based survey tool (i.e., students could not complete the survey more than once). Participants were given the opportunity to go back to change answers before final submission of the survey. The link remained open for 2 weeks, affording students the ability to complete the survey at a convenient time. A reminder email was sent to the students 1 week after the original solicitation email. Following survey completion, Qualtrics provided students with a summary of their responses for later reference during the transition-to-residency course.

### Data analysis

Survey data were exported into Microsoft Excel spreadsheets (Microsoft Corp, Redmond, WA) for analysis and was analyzed using SAS software, Version 9.4 (SAS Institute, Cary, NC). For quantitative data, proportions were reported as percentages with 95% confidence intervals (CIs), and continuous variables as medians with quartiles. Spearman’s rank-order correlation coefficient was used to assess the correlation between General Self-Efficacy Score and Intolerance of Uncertainty Scale Score. Several Chi-square tests were used to examine for any statistically significant associations between individual items on the survey. A one-sample t-test was used to compare the mean of the GSE and IUS scores to their respective national averages. An analysis of variance (ANOVA) was employed to compare average scoring across the levels within a question. Likert responses in the questionnaire were reformatted as numerical responses to take advantage of and correctly employ Chi-Square tests. All methods used in the data analyses used a critical level (alpha) of 0.05.

For qualitative data, open- and axial-coding methods were used to code individual open-ended responses, generate concepts, and organize responses into thematic categories by two study investigators using Braun and Clarke’s Thematic Analysis Six-Step Framework[23]. A third investigator reviewed the resultant themes and codes. Those with discordant interpretations were discussed until consensus was reached. The authors are cognizant that free-text survey responses do not represent rigorous qualitative research, especially when researchers attempt to address ‘how’ and ‘why’ questions[24]. This still represented an opportunity to elicit student responses about educational and curricular programs that prepared them for the uncertainty they encountered in the clinical environment. The authors also leveraged the free-text survey items as an opportunity for program improvement at their respective medical school, as new programs could be informed and developed from this data to address any gaps in the curriculum. For this reason, open-response items were limited to those that addressed ‘what’ questions (i.e., What activities do you think would better prepare you for the uncertainty in the clinical workplace?)[24].

## Results

Two hundred eighty-seven medical students completed the survey (287/290; 98.9% response rate). Student mean GSE score (31.1) was higher than the mean reported in the literature for the international community (29.6) [p < 0.001][19]. Medical student IUS mean score was not significantly different than previously cited community means (29.7 vs 29.5, respectively; p = 0.8)[21].

Student GSE and IUS scores were inversely correlated (p < 0.0001). GSE scores correlated with all Clinical Practice Uncertainty Domains (CPUDs) (p < 0.001). IUS scores inversely correlated with all CPUDs (p < 0.001), with the exception of patient communication during clinical uncertainty (p = 0.3).

Student perceptions of specific curricular programs and pedagogies correlated with CPUDs; this is summarized in Table 1. Curricular components with statistically significant correlations are tabulated.

**Table 1.** Correlations between clinical practice uncertainty domains and educational experiences among medical students

Clinical Practice Uncertainty Domain	Educational Experiences with Ratings Correlated to Clinical Practice Uncertainty Domain Ratings
Preparation	Clinical Team Debriefs (p = 0.04) Faculty facilitated peer reflection groups (p = 0.02) Case-Based Learning (p = 0.03)
Patient Communication	Talking about experiences (p = 0.03) Clinical Story Slams (p = 0.03) Required Scholarly Activity (p = 0.03)
Patient Relationships	Clinical Team Debriefs (p = 0.01) Small-Group Communication Skills Practice (p = 0.02) Talking about experiences (p = 0.01) Journaling experiences (p = 0.03)
Well-Being	Small-Group Communication Skills Practice (p = 0.02) Team-Problem Solving Sessions (p = 0.02) Writing Reflections and Narratives (p = 0.04) Journaling experiences (p = 0.02)

When asked what would prepare students for uncertainty in clinical practice, specific qualitative themes were identified (Table 2). One hundred twenty-six students submitted free responses. Students found the following experiences useful as they prepared for uncertainty in clinical practice: reflections with emotional vulnerability from instructors, small-group learning, simulations, debriefing, communication demonstrations, faculty role modeling, storytelling, and wellness prioritization. Clinical experience was the most frequently observed theme with regards to preparing for uncertainty in clinical practice.

**Table 2.** Medical student suggestions for educational experiences that would prepare them for uncertainty in clinical practice

Theme	Number of Comments	Representative Quotes *
Experience	43	‘Being in the clinic and facing problems directly is the only way.’
Reflections	34	‘It’s okay [for attendings] to have emotions and talk about those emotions, it’s okay to have a bad day, it’s okay to acknowledge when you are stressed.’ ‘I think it is super helpful to work in a team where people say out loud that there are uncertainties and address how that makes them feel.’
Small-Group Learning	17	‘More CBL [case-based learning] or TBL [team-based learning] type cases where there ISN’T a “right” answer. Even in CBLs there is always a right answer at the end and people hang on to that.’ ‘CBL cases that don’t end with a satisfying diagnosis – include lab values that don’t tell a clear story, incorporate multiple day hospital stays where not much changes into the narrative, and ultimately have the outcome be that the patient goes home stable but without a neat and tidy ending’ ‘More clinical skills small-group sessions about uncertain scenarios.’
Simulations	17	‘I think more sim cases would teach students to get out of the multiple choice thinking process, and realize that real life is much more fluid and less concrete.’ ‘Simulations where they cannot win (stop the patient from dying), like in Star Trek’
Debriefing	16	‘My greatest benefit came with debriefing an actual event with the resident who stood alongside me.’
Demonstrating Communication	11	‘Seeing good examples of physicians explaining things to patients when they don’t know exactly in a concrete way and what they DO know and what they are going to do to work to figure it out.’
Role Modeling	14	‘Seniors who demonstrate how you can tolerate it successfully, show it is not a failure, reveal how to create relationships with patients while being uncertain.’
Storytelling	10	‘More stories from more professionals.’ ‘I’m a big fan of the faculty sharing their stories with us.’
Prioritizing Wellness	9	‘Students experience incredible distress when there is not a clear-cut-and-dry answer and they likewise approach medicine from a strictly resume-padding approach (whatever gets them to honor and match into some surgical specialty, etc.).’ ‘Less emphasis on exams, as they rot our brains and turn us into robots with canned empathy.’

* Representative quotes shared above are from different medical students

The theme of reflection was observed in student comments that appreciated open dialogue with faculty and peers, where feelings associated with clinical uncertainty were acknowledged and discussed. For small-group learning, students appreciated sessions discussing unclear situations and opportunities to reflect on real-life events. For simulated encounters, students valued being forced to adapt to unexpected or unsatisfying outcomes. Clinical debriefs helped students process clinical events characterized by uncertainty. In particular, bedside debriefing with team members, either immediately following an event or at a later scheduled time, was also appreciated.

Students also valued observing examples of physicians communicating uncertainty with their patients. Specifically, students commented on how providers explained what they did not know in a concrete way, as well as what they did know about a clinical situation. They commented on the ability of role modeling to help prepare them to acknowledge unclear situations, while still forming relationships with their patients despite the uncertainty.

Students appreciated the personal narratives interprofessional providers shared with them during times of uncertainty. Students felt that these stories normalized uncertainty. Finally, students described the benefits of prioritizing wellness when reconciling uncertainty. Students felt that embracing a mindset away from living life as if there is always a ‘cut-and-dry answer’ would better prepare them for uncertainty in clinical practice.

## Discussion

Medical school curricula must introduce students to the uncertainty that exists in clinical practice to help them thrive in today’s healthcare system. Students will find themselves in ambiguous situations that will challenge their clinical reasoning skills and their confidence[25]. Students must also be equipped with the skills to openly discuss, reconstruct, and redefine their understanding of clinical problems, as they arise[10].

Research, testimonials, and recent current events have acknowledged the importance of guiding both novice and experienced physicians through the uncertainty that is replete in medicine [5,26,27]. Physicians’ mindsets about uncertainty have been shown to affect stress levels [28] and attributional styles towards certain diseases[4]. Student narratives speak to the mismatch of the clinical milieu with the medical training graduates typically receive [8,29,30]. Few studies, however, have surveyed medical students about specific pedagogies that have helped, or would have helped, them navigate this uncertainty during their clinical clerkships, their first transition into the clinical environment. To our knowledge, this is the first study to evaluate this question.

Our results demonstrated a statistically significant inverse correlation between student self-efficacy and intolerance of uncertainty, suggesting that as student self-efficacy increased, so did one’s tolerance for uncertainty. While the relationship between intolerance of uncertainty and general self-efficacy in medical education has not been clarified in the literature, a positive correlation between tolerance for ambiguity and self-efficacy has been described[15]. Self-efficacy does not always correlate with task performance[31]; however, it does correlate with decreased burnout[32], better emotional regulation, and improved academic performance due to the ability to persist in the face of difficult tasks[33]. The GSE has been criticized for its use in medical education research, as it does not have domain specificity[14]; however, in situating the scale within a survey that consisted of items relating to uncertainty in clinical practice, it is likely our students looked at self-efficacy from this lens. Nonetheless, given this observation in the data, curriculum developers should incorporate programs that build student self-efficacy during medical school training to prepare them for the transition into clinical practice.

Our results support that curriculum developers should deliberately include conversations surrounding uncertainty in a medical school curriculum. Our data suggests that students want to openly discuss and debrief experiences that were characterized as uncertain – from real experiences in the clinical environment, to artificial scenarios recreated in a simulation laboratory. Students expressed an interest in additional experiences that would support productive struggle during uncertain situations – experiences that could easily be built into a curriculum through simulation-mediated modalities. Students particularly expressed an interest in exploring this uncertainty with faculty who have experienced it through role-modeling. Imparting lessons on effectively navigating uncertainty in clinical practice can be well-facilitated through role-modeling, and actively involves the student in deciding whether or not to trial specific behaviors observed and/or discussed[34].

One possible solution to formally include these conversations in a curriculum is through case-based learning, where there are multiple possible ways to address a clinical encounter with a variety of case endings[35], or when no specific diagnosis is reached. A statistically significant relationship was observed for students’ perceptions of CBL’s role in preparing them for uncertainty in clinical practice. Similarly, student comments highlighted the role small-group learning, such as CBL, can have as a curricular approach to prepare them for clinical uncertainty. CBL allows students to struggle with complex problems, which can help lead to more durable and flexible learning in the long-term. Struggle, failure, and problem-solving can help build a foundation in learners to use acquired knowledge to generate solutions in new contexts[36], such as when encountering uncertain problems in the CLE.

Formally learning how to facilitate debriefings can provide students with the skills to lead these discussions during times of uncertainty in the clinical environment[37]. To increase comfort with diagnostic uncertainty, students can be introduced to frameworks that can help scaffold conversations with patients. One-third of patients discharged from the emergency department (ED) do not receive a diagnosis to explain their symptoms, yet there is no established approach for effective discharge communication during these instances[22]. Rising et al have introduced the uncertainty communication checklist (UCC) to scaffold conversations when discharging patients from the emergency department (ED) with diagnostic uncertainty [22,38]. Practicing the UCC with students in small group, or as objective structured clinical examinations (OSCEs), for example, would allow students to better learn how to communicate uncertainty, and can serve as a guide to navigate communication with patients. Similarly, including conversations around shared decision-making can further prepare students for speaking with their patients during times of uncertainty[39].

A survey of students from five US medical schools showed that exposure to a humanities curriculum was significantly correlated with tolerance for ambiguity[15]. Based on this study, we would expect that a humanities curriculum correlates well with students’ self-reported preparedness for uncertainty across different domains. As expected, many of the educational programs that correlated with uncertainty practice domains in our survey were specific humanities sessions. These sessions, which were not explicitly labeled as humanities sessions, were typically highly rated; however, when humanities as a whole was specifically asked about, statistically-significant correlations were not observed in the data in contrast to the study by Mangione et al [15]. This difference may be due to the fact that our medical school offers a diverse offering of sessions within its required humanities curriculum so students received varied content. We did not survey students on every element of the humanities curriculum, as the sample size for each individual element would have been too small to detect statistically-significant relationships.

The role of uncertainty in burnout also merits consideration. Higher IUS scores have been shown to correlate with burnout in Australian general practice registrars[40]. In their study, Cooke et al found that a score of 36.6 ± 9.8 was correlated with high burnout and a score of 30.2 ± 7.2 was correlated with low burnout[40]. Our students’ scores were similar to that of the general population, and were lower on average than scores in the Australian study. A recent systematic review noted that there appears to be a relationship between tolerance of ambiguity and psychological well-being in medical training[41]. Given that the concept of self-efficacy seems to be correlated with tolerance for uncertainty, it is suggested that any attempt to improve student wellbeing should include developing familiarity with uncertainty through deliberate exposure, practice, debriefing, and role-modeling. It is common for wellness to imply one’s personal wellness and resilience; however, curricular changes to allow for ample discussion of medical school experiences can also serve as a forum to foster wellness and well-being.

There are several limitations worth noting. Our data reflects student experiences from a single urban medical school situated within a large academic medical center with multiple hospital affiliations. This may have implications on the generalizability of our findings. Furthermore, our students complete their clinical rotations at various hospitals within our system, each of which can differently influence student perceptions of uncertainty in the clinical learning environment. Our team did not examine student responses by clinical site. Additionally, the effects of recall bias should be considered, as students were asked to cite pre-clinical experiences that took place in the curriculum more than one year prior to being surveyed.

While the survey consisted of two validated instruments, along with additional items that were piloted by the study investigators prior to survey dissemination, the composite survey was not validated. With regards to general self-efficacy and intolerance of uncertainty, student data was compared to normative groups for the GSE and IUS instruments, respectively [19,21]. These normative groups may not be representative of our student body for valid comparisons. The instrument itself consisted of thirty items and was lengthy in nature. Although our response rate was close to 99% with several statistically significant relationships observed, there is a likelihood of influencing factors from survey fatigue.

Furthermore, the data for all participants who completed the questionnaire was analyzed as a whole. The authors did not examine for any differences between students (i.e., students who identify as female versus students who identify as male). While this would have likely yielded interesting results, the authors made a deliberate decision to take a more holistic approach and examine the experiences of the collective. Follow-up studies should include sub-group analyses to detect differences across students. Similarly, follow-up studies could examine differences across students pursuing different specialties. In a pilot study with preliminary data, the authors found that specialty choice did not correlate with intolerance of uncertainty and general self-efficacy given small sample sizes across sub-groups. Subsequent studies should be powered with sample sizes large enough to detect differences. There is an opportunity to expand this investigation to other medical schools and institutions to not only adequately power follow-up studies for sub-group analyses, but to also determine how these correlations stand in larger groups of medical students.

## Conclusion

The relationships and themes from this study can be used to inform hypothesis generation regarding the types of educational activities educators can consider when preparing students for clinical uncertainty. Our data suggests that strategically integrating educational modalities that explicitly address uncertainty during pre-clerkship training may better prepare students for the uncertainty ubiquitous in clinical practice. Preparation for uncertainty in clinical practice may improve student self-efficacy in the face of this uncertainty and enhance student well-being. Based on this study, clinical debriefs, interprofessional role playing, simulations, communications skills sessions, instructor emotional vulnerability, storytelling, and peer-to-peer conversations may have the most impact. Additional research to evaluate the impact of these interventions is needed.

## Appendix (Survey)Uncertainty in Clinical Practice

**Start of Block: Self-Efficacy Scale**

Dear students, We hope this email finds all of you well.

These are unprecedented times. Now, more than ever, is the notion of uncertainty critical. While the evolving nature of the COVID-19 pandemic remains steeped with uncertainty, your professional careers – regardless of the specialty you choose to pursue – will be complicated by uncertainty. Down the road, you are sure to encounter uncertainty when gathering information; making decisions; identifying a patient’s diagnosis; working with a new team; encountering new epidemics and crises; and/or admitting that you do not know what to do next during a specific clinical scenario. This is normal. Some of us may be comfortable with this uncertainty. Most of us, however, find this as a source of stress. The goal of this session is to introduce uncertainty as a ‘character’ in our professional narratives. We hope that by speaking about it and putting a name to it, we can become more comfortable on what to do when we encounter uncertainty in clinical practice. To help you reflect on this topic, we ask that you complete the attached survey. You are being asked to complete the Intolerance of Uncertainty Scale and the General Self-Efficacy Scale. Please review your results and consult the attached reading to better understand what your relationship is with uncertainty. You will also notice several questions that ask about the impact of the school’s curriculum on your ability to tolerate uncertainty in your clinical practice. Thank you for your time. We hope you and your families are well and safe. Please do not hesitate to contact us.

**PART 1: The General Self-Efficacy Scale**Self-efficacy is a measure of one’s confidence in her/his ability to act in a particular situation. The following scale was developed to evaluate one’s coping ability in daily living. **Responses are anonymous. Please reflect honestly rather than selecting what you think should be the right answer.**

Q1 I can always manage to solve problems if I try hard enough.

1. 1 = Not at all true (1)
2. 2 = Hardly true (2)
3. 3 = Moderately true (3)
4. 4 = Exactly true (4)

Q2 If someone opposes me, I can find the means and ways to get what I want.

1. 1 = Not at all true (1)
2. 2 = Hardly true (2)
3. 3 = Moderately true (3)
4. 4 = Exactly true (4)

Q3 It is easy for me to stick to my aims and accomplish my goals.

1. 1 = Not at all true (1)
2. 2 = Hardly true (2)
3. 3 = Moderately true (3)
4. 4 = Exactly true (4)

Q4 I am confident that I could deal efficiently with unexpected events.

1. 1 = Not at all true (1)
2. 2 = Hardly true (2)
3. 3 = Moderately true (3)
4. 4 = Exactly true (4)

Q5 Thanks to my resourcefulness, I know how to handle unforeseen situations.

1. 1 = Not at all true (1)
2. 2 = Hardly true (2)
3. 3 = Moderately true (3)
4. 4 = Exactly true (4)

Q6 I can solve most problems if I invest the necessary effort.

1. 1 = Not at all true (1)
2. 2 = Hardly true (2)
3. 3 = Moderately true (3)
4. 4 = Exactly true (4)

Q7 I can remain calm when facing difficulties because I can rely on my coping abilities.

1. 1 = Not at all true (1)
2. 2 = Hardly true (2)
3. 3 = Moderately true (3)
4. 4 = Exactly true (4)

Q8 When I confronted with a problem, I can usually find several solutions.

1. 1 = Not at all true (1)
2. 2 = Hardly true (2)
3. 3 = Moderately true (3)
4. 4 = Exactly true (4)

Q9 If I am in trouble, I can usually think of a solution.

1. 1 = Not at all true (1)
2. 2 = Hardly true (2)
3. 3 = Moderately true (3)
4. 4 = Exactly true (4)

Q10 I can usually handle whatever comes my way.

1. 1 = Not at all true (1)
2. 2 = Hardly true (2)
3. 3 = Moderately true (3)
4. 4 = Exactly true (4)

**End of Block: Self-Efficacy Scale**

**Start of Block: Intolerance to Uncertainty**

**PART 2: Intolerance of Uncertainty Scale** Intolerance of uncertainty represents an individual’s negative beliefs about uncertainty and its implications. The Intolerance of Uncertainty Scale helps quantify the beliefs we have about uncertainty in life. Responses are anonymous.Please reflect honestly rather than selecting what you think should be the right answer.

Q15 Unforeseen events upset me greatly.

1. 1 = not at all characteristic of me (1)
2. 2 = a little characteristic of me (2)
3. 3 = somewhat characteristic of me (3)
4. 4 = very characteristic of me (4)
5. 5 = entirely characteristic of me (5)

Q16 It frustrates me not having all the information I need.

1. 1 = not at all characteristic of me (1)
2. 2 = a little characteristic of me (6)
3. 3 = somewhat characteristic of me (7)
4. 4 = very characteristic of me (8)
5. 5 = entirely characteristic of me (9)

Q17 Uncertainty keeps me from living a full life.

1. 1 = not at all characteristic of me (1)
2. 2 = a little characteristic of me (6)
3. 3 = somewhat characteristic of me (7)
4. 4 = very characteristic of me (8)
5. 5 = entirely characteristic of me (9)

Q18 One should always look ahead so as to avoid surprises.

1. 1 = not at all characteristic of me (1)
2. 2 = a little characteristic of me (6)
3. 3 = somewhat characteristic of me (7)
4. 4 = very characteristic of me (8)
5. 5 = entirely characteristic of me (9)

Q19 A small unforeseen event can spoil everything, even with the best of planning.

1. 1 = not at all characteristic of me (1)
2. 2 = a little characteristic of me (6)
3. 3 = somewhat characteristic of me (7)
4. 4 = very characteristic of me (8)
5. 5 = entirely characteristic of me (9)

Q20 When it’s time to act, uncertainty paralyzes me.

1. 1 = not at all characteristic of me (1)
2. 2 = a little characteristic of me (6)
3. 3 = somewhat characteristic of me (7)
4. 4 = very characteristic of me (8)
5. 5 = entirely characteristic of me (9)

Q21 When I am uncertain I can’t function very well.

1. 1 = not at all characteristic of me (1)
2. 2 = a little characteristic of me (6)
3. 3 = somewhat characteristic of me (7)
4. 4 = very characteristic of me (8)
5. 5 = entirely characteristic of me (9)

Q22 I always want to know what the future has in store for me.

1. 1 = not at all characteristic of me (1)
2. 2 = a little characteristic of me (6)
3. 3 = somewhat characteristic of me (7)
4. 4 = very characteristic of me (8)
5. 5 = entirely characteristic of me (9)

Q23 I can’t stand being taken by surprise.

1. 1 = not at all characteristic of me (1)
2. 2 = a little characteristic of me (6)
3. 3 = somewhat characteristic of me (7)
4. 4 = very characteristic of me (8)
5. 5 = entirely characteristic of me (9)

Q24 The smallest doubt can stop me from acting.

1. 1 = not at all characteristic of me (1)
2. 2 = a little characteristic of me (6)
3. 3 = somewhat characteristic of me (7)
4. 4 = very characteristic of me (8)
5. 5 = entirely characteristic of me (9)

Q25 I should be able to organize everything in advance.

1. 1 = not at all characteristic of me (1)
2. 2 = a little characteristic of me (6)
3. 3 = somewhat characteristic of me (7)
4. 4 = very characteristic of me (8)
5. 5 = entirely characteristic of me (9)

Q26 I must get away from all uncertain situations.

1. 1 = not at all characteristic of me (1)
2. 2 = a little characteristic of me (6)
3. 3 = somewhat characteristic of me (7)
4. 4 = very characteristic of me (8)
5. 5 = entirely characteristic of me (9)

**End of Block: Intolerance to Uncertainty**

**Start of Block: Block 2**

**PART 3: Uncertainty in Clinical Practice** Please reflect on your experiences during clinical clerkships. The following questions focus on how you were affected by uncertainty in the clinical environment. Responses are anonymous. Please reflect honestly rather than selecting what you think should be the right answer.

Q27 **I feel prepared to address uncertain situations during clinical clerkships**. Several examples of uncertain situations may include: What do I do when I care for a patient with an unclear diagnosis? How do I choose the right treatment option when I cannot control my patient’s social determinants of health? What do I do when I do not know the answer to a patient’s question?

1. Not at all (1)
2. Somewhat (3)
3. Very (4)
4. Entirely (6)

Q32 **I am confident in my ability to communicate to patients during clinical situations that may be uncertain**. Clinical examples may include discussions about prognosis; medication side effects; discharge plans; conversations with family members.

1. Not at all (1)
2. Somewhat (6)
3. Very (7)
4. Entirely (8)

Q34 **When I encounter clinically uncertain situations, I am still able to form meaningful relationships with my patients**.

1. Not at all (1)
2. Somewhat (6)
3. Very (7)
4. Entirely (8)

Q33 **When I encounter clinically uncertain situations, my well-being is negatively affected**.

1. Not at all (1)
2. Somewhat (6)
3. Very (7)
4. Entirely (8)

**End of Block: Block 2**

**Start of Block: Educational tools and programs**

Q44 **PART 4: Medical School and Preparedness for Clinical Uncertainty** Q29 Please review the following experiences the curriculum has offered you. Indicate how the following experiences have prepared you to address uncertainty in the clinical environment.

**Table:**

	None at all (55)	A little (56)	A moderate amount (57)	A lot (58)	A great deal (59)
Case Based Learning (CBL) (1)					
Clinical Skills Small Group (CSSG) (2)					
TBL (17)					
Clinical Experience (CE) (18)					
Skills Observation and Reflection Groups (SOAR) (3)					
Interprofessional programs (e.g., Health Mentors, TeamSAFE) (15)					
Scholarly Inquiry (13)					
Humanities (4)					
Writing reflections and narratives (14)					
Class lectures (16)					

Q41 Please review the following electives/activities that may have been offered to you while in medical school. Indicate how the following experiences have prepared you to address uncertainty in the clinical environment.

**Table:**

	Did not participate (46)	None at all (41)	A little (42)	A moderate amount (43)	A lot (44)	A great deal (45)
Story Slams (5)						
Failing Forward (17)						
Mindfulness activities (7)						
Clinical team debriefs (12)						
Talking about my clinical experiences with others (peer narratives) (8)						
Journaling my experiences (14)						
Faculty Narratives (16)						

Q37 What tools or activities do you think would help students prepare for clinical uncertainty?

________________________________________________________________
